# Aptamers and Aptamer-Coupled Biosensors to Detect Water-Borne Pathogens

**DOI:** 10.3389/fmicb.2021.643797

**Published:** 2021-02-19

**Authors:** Mariam Saad, Sebastien P. Faucher

**Affiliations:** ^1^Department of Natural Resources, Faculty of Agricultural and Environmental Sciences, McGill University, Montreal, QC, Canada; ^2^Centre de Recherche en Infectiologie Porcine et Avicole (CRIPA), Université de Montréal, Faculté de Médecine Vétérinaire, Saint-Hyacinthe, QC, Canada

**Keywords:** aptamer, SELEX, water-borne pathogens, viable but non-culturable, coliforms, aptasensors

## Abstract

Aptamers can serve as efficient bioreceptors for the development of biosensing detection platforms. Aptamers are short DNA or RNA oligonucleotides that fold into specific structures, which enable them to selectively bind to target analytes. The method used to identify aptamers is Systematic Evolution of Ligands through Exponential Enrichment (SELEX). Target properties can have an impact on aptamer efficiencies. Therefore, characteristics of water-borne microbial targets must be carefully considered during SELEX for optimal aptamer development. Several aptamers have been described for key water-borne pathogens. Here, we provide an exhaustive overview of these aptamers and discuss important microbial aspects to consider when developing such aptamers.

## Introduction

Access to water that is safe for use and consumption is a basic human right. As a result, most countries have strict guidelines, regulations and standards for managing water sources and water distribution systems to supply high quality water free from chemical and microbial contaminants. In most cases, microbial contaminants must be removed from the water before distribution. These microbes include pathogens that cause gastroenteritis, such as *Cryptosporidium, Giardia, Norovirus, Rotavirus, Campylobacter, and E. coli* (WHO, 2017). Other water-borne diseases are caused by pathogens growing inside water distribution systems or within engineered water systems, such as cooling tower, fountains, spas and humidifiers (Wang H. et al., 2017). The latter include *Legionella pneumophila*, *Pseudomonas* spp. and non-tuberculosis mycobacteria. In recent years, several studies have shown that a high proportion of water associated deaths and illnesses are due to the aforementioned three environmental water-borne pathogens (Gargano et al., 2017; Greco et al., 2020). In fact, *L. pneumophila*, the causative agent of Legionnaires disease, has become the number one cause of water-borne outbreaks in recent years (McClung et al., 2017). The presence of coliforms is not indicative of the presence of several key water-based pathogens that are of significance to public health (Payment and Locas, 2011). Consequently, specific detection methods are needed to ensure safe water from the source to the point of use.

Monitoring and surveillance of specific water-borne microbes require robust detection methods. Challenges in select current detection methods for waterborne pathogens have been reviewed excellently in detail elsewhere (Ramírez-Castillo et al., 2015; Wang H. et al., 2017). In general, traditional microbial detection methods rely heavily on culture methods, which is fraught with several limitations. Culture methods are extremely time consuming and often require extensive material, specialized labor, and time. Culture recovery rates are also adversely affected by many factors such as the presence of competing microbes, the presence of viable but non-culturable (VBNC) cells, methods used for concentration of the sample or enrichment of the target microbe and sample type (bulk water or biofilm) (Wang H. et al., 2017). Drawbacks with culture techniques has led to a shift toward the use of molecular methods, including PCR, quantitative PCR (qPCR), high throughput sequencing, and immunoassays such as ELISA, immunochromatography and immuno-lateral flow assays. The most widely used molecular method is qPCR (Ramírez-Castillo et al., 2015; Wang H. et al., 2017). The advantage of qPCR, over conventional culture techniques, is more rapid turn-around times, high sensitivities and specificities, lower limits of detection, as well as an ability to detect VBNC cells. However, by detecting live, VBNC and dead cells qPCR leads to an overestimation of microbial burden. Additionally, qPCR involves multiple sample processing steps which requires specialized labor. qPCR is also inhibited by several compounds routinely found in water samples resulting in possible false negatives (Gentry-Shields et al., 2013).

Biosensors can mitigate some of the problems associated with traditional detection methods (Ahmed et al., 2014). They are analytical devices used to quantify or detect a specific analyte (Turner, 2013). Qualities of biosensors includes high specificity, high sensitivity, multiplexing capability, cost-effectiveness, portability and ease of use (Ahmed et al., 2014; Kumar et al., 2018; Cesewski and Johnson, 2020; McConnell et al., 2020). A biosensor set-up typically consists of three elements. A biorecognition element, which upon interaction with a target, produces a physico-chemical signal that is converted by a transducing element into a signal captured by a detection element (Turner, 2013). Biosensors are categorized based on either their transducing element (mechanical, optical, electrochemical) or the nature of the biorecognition element (affinity, catalytic) (Ahmed et al., 2014).

A versatile and stable biorecognition element is a critical component of any biosensing platform (Ahmed et al., 2014; Kumar et al., 2018). Antibodies are the most used bioreceptors in biosensor development and research, but aptamers are an increasingly widespread popular alternative (Song et al., 2008; Morales and Halpern, 2018). Aptamers are single stranded DNA or RNA oligonucleotides that fold into specific complex structures and interact with their targets via shape complementarity, hydrogen bonding, electrostatic interactions and stacking interactions (McKeague et al., 2015). Besides having high affinities and selectivity, they can bind to a wide range of targets from small non-immunogenic compounds to whole cells (McKeague et al., 2015). Aptamers can be generated *in vitro* in conditions one can preferentially select making them stable and versatile for a variety of applications (Song et al., 2008). They are cost-effective to synthesize with minimal batch to batch variation (Strehlitz et al., 2012; McConnell et al., 2020). Their easily modifiable nature facilitates functionalization on sensing surfaces (Song et al., 2008; McConnell et al., 2020). Their inherent small size also promotes high packing densities during functionalization (Song et al., 2008; Crivianu-Gaita and Thompson, 2016). In this minireview, we will briefly provide examples of aptamers with potential for detection of water-borne pathogens and discuss microbial determinants for the development of optimal aptamers and thus improved aptamer-coupled biosensors. Examples of aptamers is provided in Table 1 and a complete list of aptasensing platforms is provided in Supplementary Table 1.

**TABLE 1:** Aptamers developed against water-borne bacteria.

**Aptamer name**	**Target**	**Culture condition^*a*^**	**OD/Growth stage^*a,b*^**	**Counter-Selex Strains^*c*^**	**Type of sensors**	**LOD**	**References**
***Norovirus***							
AG3	MuNoV	*NA*	*NA*	Feline calicivirus (FCV)	Electrochemical	180 virus particles	Giamberardino et al., 2013
				*NA*	Optical (colorimetric)	200 virus/ml	Weerathunge et al., 2019
Aptamer 25/SMV-25	SMV	*NA*	*NA*	HuNoV-negative human stool suspension, bead-antibody complex	*NA*	*NA*	Escudero-Abarca et al., 2014
	Non-toxic norovirus GII capsid recombinant			*NA*	Optical (Chemiluminescence)	80 ng/ml	Kim B. et al., 2018
Aptamer 21/SMV-21	SMV	*NA*	*NA*	HuNoV-negative human stool suspension, bead-antibody complex	*NA*	*NA*	Escudero-Abarca et al., 2014
	Norovirus Group II (recombinant VLP)			*NA*	Electrochemical	100 pM	Chand and Neethirajan, 2017
***C. parvum***							
R4-6	Oocysts	*NA*	*NA*	*Giardia duodenalis* cysts	Electrochemical	100 oocysts	Iqbal et al., 2015
				*NA*	Electrochemical	50 oocysts	Iqbal et al., 2019
Min_Crypto2	Oocysts	*NA*	*NA*	*NA*	Optical fluorescence	5 oocysts	Hassan et al., 2021
***Acinetobacter***							
Aci49	Whole-cell-*A. baumannii* (ATCC 19606)	BHI broth, 37°C, overnight	0.4/E	*Acinetobacter lwoffii, Acinetobacter calcoaceticus*, and 11 species	Optical (colorimetric)	10^3^ CFU/ml	Rasoulinejad and Gargari, 2016
				*NA*	Optical (fluorescence)	3 CFU/ml	Li et al., 2020
				*NA*	Optical (fluorescent)	10 CFU/ml	Yang et al., 2020
AB aptamer	Whole-cell *A. baumannii*	**NR**	**NR**	**NR**	Optical (colorimetric)	450 CFU/rxn	Wu et al., 2018
				*NA*	Optical (fluorescence)	10^5^ CFU/ml	Su et al., 2020
				*NA*	Optical (fluorescence)	100 CFU/ml	Su et al., 2020
				*NA*	Optical (fluorescence)	300 CFU/ml	Bahari et al., 2021
***Aeromonas***							
Apt1	Whole-cell (*A. hydrophila)*	LB,37°C, 18 h	**NR**	**NR**	Optical (Fluorescence)	1.5 CFU/ml	Zhu et al., 2019
***Campylobacter***							
Aptamer C2 and Aptamer C3	Surface protein (*C. jejuni*)	**NR**	**NR**	**NR**	Optical (fluorescence)	2.5 CFU/ml	Bruno et al., 2009
				*NA*	Optical (colorimetric)	5–10 CFU/ml	Bruno and Sivils, 2017
ONS-23	Whole-cell (*C. jejuni* A9a)	BBL brucella broth, 42°C, 48 h, microaerophillic conditions	PE*	20 strains (enteric, non-enteric, lactic acid)	*NA*	*NA*	Dwivedi et al., 2010
				*NA*	Optical (colorimetric)	10 CFU/ml	Dehghani et al., 2018
				*NA*	Optical (colorimetric)	7.2 × 10^5^ CFU/ml	Kim Y. J. et al., 2018
CJA1	Whole-cell (*C. jejuni*)			**NR**	Optical (colorimetric)	10 CFU/ml	Chen et al., 2020
***Cyanobacteria***							
ATX8	Anatoxin-a (ATX)	*NA*	*NA*	ATX free beads	Electrochemical	0.5 nM	Elshafey et al., 2015
MC-LR aptamer/AN6	Microcystin-LR	*NA*	*NA*	Blank sepharose beads	Electrochemical	10 pM	Ng et al., 2012
				*NA*	Optical (fluorescence)	0.002 ng/ml	Lv et al., 2017
***E. coli***							
L9F	O111-LPS (*E. coli* O111:K58)	35°C, TSB, overnight	**NR**	**NR**	*NA*	*NA*	Bruno et al., 2008
				*NA*	Electrochemical	112 CFU/ml	Luo et al., 2012
Eco4R/ECAII	Outer membrane protein (OMP)—*E. coli* 8739	37°C, blood agar, overnight	**NR**	**NR**	*NA*	*NA*	Bruno et al., 2010
				*NA*	Electrochemical	**NR**	Queirós et al., 2013
Eco4F	OMP-*E. coli* 8739	37°C, blood agar, overnight	**NR**	**NR**	*NA*	*NA*	Bruno et al., 2010
				*NA*	Optical (colorimetric/fluorescence)	300 CFU/ml	Bruno, 2014
Eco3R/ECAI	OMP-*E. coli* 8739	37°C, blood agar, overnight	**NR**	**NR**	*NA*	*NA*	Bruno et al., 2010
				*NA*	Electrochemical	**NR**	Queirós et al., 2013
				*NA*	Optical (colorimetric/fluorescence)	300 CFU/ml	Bruno, 2014
				*NA*	Optical (Evanescent wave fiber optics)	0.1nM	Queirós et al., 2014
E1	Whole cell (*E. coli* fecal isolate)	NB, 37°C	0.45/E	*E. coli* (non-fecal isolate), other fecal isolates	*NA*	*NA*	Kim et al., 2013
E2	Whole cell (*E. coli* fecal isolate)	NB, 37°C	0.45/E	*E. coli* (non-fecal isolate), other fecal isolates	*NA*	*NA*	Kim et al., 2013
				*NA*	Optical (fluorescence)	3 CFU/ml	Jin et al., 2017
				*NA*	Electrochemical	100 CFU/ml	Wu et al., 2017
E10	Whole cell (*E. coli* fecal isolate)	NB, 37°C	0.45/E	*E. coli* (non-fecal isolate), other fecal isolates	*NA*	*NA*	Kim et al., 2013
E1 + E2 + E10 (pooled)				*NA*	Electrochemical	371 CFU/ml	Kim et al., 2014
AptB12	Whole cell (*E. coli* ETEC K88)	LB	E	ETEC K99, *S. enteritidis, S. aureus*,	Optical (fluorescence)	1.1 × 10^3^ CFU/ml	Peng et al., 2014
RNAaptamer	**NR**	LB, 37°C, 2–3 h	**NR**	*NA*	Electrochemical	**NR**	So et al., 2008
				*NA*	Immunomagnetic separation and RT-PCR	10 CFU/ml	Lee et al., 2009
				*NA*	Electrochemical	6–26 CFU/ml	Zelada-Guilleìn et al., 2010
Aptamer I-1	O-antigen LPS (*E. coli* O157:H7)	Brucella broth,37°C,24 h (+0.04% formaldehyde)	**NR**	*E. coli* K12	*NA*	*NA*	Lee et al., 2012
				*NA*	Electrochemical	4 CFU/ml	Burrs et al., 2016
Ec3 (31)	Whole cell (*E. coli* DH5α)	LB	0.4	*B. subtilis*	Electrochemical	2 × 10^4^ CFU/ml	Dua et al., 2016
P12-31	Whole cell (*E. coli* O6)	37°C, LB	0.3	**NR**	*NA*	*NA*	Marton et al., 2016
AM-6	Whole cell (*E. coli* O157:H7)	LB	0.6	*E. coli* strains O42, K12, Top10, DH5α, *S. flexneri, S. Typhi*	*NA*	*NA*	Amraee et al., 2017
S1	Whole cell (*E. coli* O157:H7)	BHI, 37°C	E	*S. aureus*, *S. Typhyimurium*, *L. monocytogens*	Mechanical (Quartz Crystal Microbalance-QCM)	1.46 × 10^3^ CFU/ml	Yu et al., 2018
Apt-5	whole cell (*E. coli* O157:H7)	LB, 37°C	**NR**	*E. coli* ETEC and 3 other species	*NA*	*NA*	Zou et al., 2018
a-aptamer/E-17F72*	O157:H7 LPS	LB, 37°C	**NR**	**NR**	*NA*	*NA*	Bruno et al., 2009
c-aptamer/E-18R72*	O157:H7 LPS	LB, 37°C	**NR**	**NR**	*NA*	*NA*	Bruno et al., 2009
a-aptamer, c-aptamer				*NA*	Optical (colorimetric)	10 CFU/ml	Wu et al., 2015
a-aptamer, c-aptamer				*NA*	Optical (colorimetric)	25 CFU/ml	Díaz-Amaya et al., 2019b
a-aptamer, c-aptamer				*NA*	Optical (surface enhanced raman spectroscopy-SERS)	100 CFU/ml	Díaz-Amaya et al., 2019a
c-aptamer				*NA*	Optical (fluorescence)	100 CFU/ml	Hao et al., 2019
				*NA*	Optical (fluorescence)	80 CFU/ml	Jiang et al., 2020
***Helicobacter pylori***							
Hp-Ag aptamer	Recombinant Hp surface antigen	**NR**	**NR**	BSA	*NA*	*NA*	Gu et al., 2018
Hp4	Recombinant Hp surface antigen	Blood agar, 37°C, 3 days	**NR**	BSA	*NA*	*NA*	Yan et al., 2019
***Legionella***							
R10C5, R10C1	Whole cell (*Lp* 120292)	CYE agar plate, 37°C, 3 days followed by AYE media,37°C, 24 h	2.5/PE	*Pseudomonas putida* KT2440, *Pseudomonas fluorescens* LMG1794	*NA*	*NA*	Saad et al., 2020
***NTM***							
BM2/N31	ManLAM, *M. bovis* (BCG)	L-J medium	E	**NR**	Optical (ELONA)	10^4^ CFU/ml	Sun et al., 2016
					Electrochemical	**NR**	Sodia et al., 2020
***Pseudomonas aeruginosa***							
F23	Whole cell (*P. aeruginosa* clinical isolate)	Mueller-Hinton (MH) media, 37°C, 24 h	**NR**	*S. maltophilia, A. baumannii*	Optical (fluorescence)	**NR**	Wang et al., 2011
				*NA*	Optical (fluorescence)	100 CFU/ml	Gao et al., 2018
				*NA*	Optical (Long range Surface Plasomon Resonance-LSPR)	1 CFU/ml	Hu et al., 2018
				*NA*	Optical (Fluorescence)	1 CFU/ml	Zhong et al., 2018
				*NA*	Electrochemical and Optical (colorimetric)	60 CFU/ml	Das et al., 2019
				*NA*	Electrochemical	33 CFU/ml	Roushani et al., 2019
				*NA*	Mechanical (piezoelectric quartz crystal)	9 CFU/ml	Shi et al., 2019
St17Lp21, St21Lp17, St08Lp17	Biofilm-derived whole cells (PA 692/ATCC 14502)	LB broth, 37°C, 16 h followed by 22°C, 42 h to make biofilm.	E	**NR**	*NA*	*NA*	Soundy and Day, 2017
F23 + St08Lp17 (pool)				*NA*	Optical (Fluorescence)	1 CFU/ml	Zhong et al., 2020
***Salmonella***							
Aptamer 33	OMP (*S. tyhpimurium* PT10)	BHI, 37°C, 2–3 h		*E. coli* OMP and LPS, *Salmonella* LPS	Magnetic bead based pull down assay and qPCR	10–100 CFU/ml	Joshi et al., 2009
				*NA*	Optical (Fluorescence)	5 CFU/ml	Duan et al., 2012
				*NA*	Electrochemical	3 CFU/ml	Ma et al., 2014
				*NA*	Electrochemical	55 CFU/ml	Hasan et al., 2018
				*NA*	Optical (LSPR)	30 CFU/ml	Yoo et al., 2015
				*NA*	Optical (LSPR)	10^4^ CFU/ml	Oh et al., 2017
ST2P	Whole cell (*S. typhimurium* ATCC 50761)	BBL-BHI, 37°C, overnight	0.3/E	*L. monocytogenes, E. coli, S. aureus, S. pneumoniae, V. parahemolyticus, C. sakazakii*	Optical (fluorescence)	25 CFU/ml	Duan et al., 2013b
				*NA*	Optical (Colorimetric, SERS)	10 CFU/ml	Duan et al., 2016
				*NA*	Optical (Fluorescence)	25 CFU/ml	Duan et al., 2014
S8-7	Whole cell (*S. typhimurium* S913)	TSB-amp, 37C, overnight	**NR**	*L. monocytogenes* Scott A *E. coli O157: H7, B. cereus, E. faecalis*	*NA*	*NA*	Dwivedi et al., 2013
C4	Whole cell (*S. typhimurium*)	BHI, 35°C, overnight	**NR**	*E. coli, S. enteritidis, S. aureus*	*NA*	*NA*	Moon et al., 2013
Apt22	Whole cell (*S. paratyphi* A)	NB,37C	2.1/E	*S. Enteritidis, S. Typhimurium, S. Cholerasuis, S. Arizonae*	Optical (chemiluminescence)	1000 CFU/ml	Yang et al., 2013
S25	Whole cell (*S. enteriditis*-multiple)	TSB, overnight	**NR**	*Salmonella* serovars-multiple			Hyeon et al., 2012
SAL26	Whole cell (*S. typhimurium* ATCC14028)	TSB,37°C, overnight culture followed by TSB,37°C, 3 h then fixing with methanol	E	4 *Salmonella enterica* serovars and 9 bacterial species.	Optical (Colorimetric)	100 CFU/ml	Lavu et al., 2016
SAL1	Whole cell (*S. paratyphi*-A ATCC 9150)	LB broth, 37°C	E	*S. Typhimurium, S. flexneri, E. coli O157:H7, Yersinia enterocolitica*	Optical (fluorescence)	10 CFU/ml	Rm et al., 2020
B5	Whole cell (*S. typhimurium* ATCC14028)	BHI broth, 37°C	PE	*S. aureus, L. monocytogenes, E. coli O157:H7*	Mechanical (QCM)	1,000 CFU/ml	Wang L. et al., 2017
***Shigella***							
Aptamer S 1	Whole cell (*Shigella dysenteriae*)	LB	E	*S. aureus, S. typhimurium, E. coli, L. monocytogenes, V. parahaemolyticus*	Optical (Fluorescence)	50 CFU/ml	Duan et al., 2013a
				*NA*	Electrochemical	1 CFU/ml	Zarei et al., 2018
Sp1	Whole cell (*Shigella sonnei* ATCC 51334)	LB, 37°C, overnight	**NR**	*S. dysenteriae, S. flexneri, S. boydii, S. typhimurium, E. coli*	Optical (fluorescence)	30 CFU/ml	Gong et al., 2015
				*NA*	Optical (SERS)	10 CFU/ml	Wu et al., 2020
Sp20	Whole cell (*Shigella sonnei* ATCC 51334)	LB, 37°C, overnight	**NR**	*S. dysenteriae, S. flexneri, S. boydii, S. typhimurium and E. coli*	Optical (Fluorescence)	30 CFU/ml	Gong et al., 2015
*S. flexneri* aptamer1	Whole cell (*Shigella flexneri*)		**NR**	**NR**	Optical (fluorescence)	100 CFU/ml	Zhu et al., 2015
SS-3, SS-4	Whole cell (*Shigella sonnei*)	NB, 37°C	**NR**	*E. coli*	Optical (Fluorescence)	1,000 CFU/ml	Song et al., 2017
*S. flexneri* aptamer‘	Whole cell (*Shigella flexneri* ATCC 12022)	NB, 37°C, 12 h	**NR**	**NR**	Optical (colorimetric)	80 CFU/ml	Feng et al., 2019
***Vibrio cholerae***							
CT916	Cholerae toxin	*NA*	*NA*	Ethanolamine-blocked magnetic beads	Optical (colorimetric)	2.1 ng/ml	Frohnmeyer et al., 2018
				*NA*	Optical (colorimetric)	1–100 ng/ml	Frohnmeyer et al., 2019
	Whole cell (*V. cholerae* O1 -Inaba, ATCC 39315 and Ogawa)	LB broth, 37°C	0.4/E	*E. coli O157:H7, S. a dysenteriae, S. enteritidis, S. Typhimurium, Yersinia* spp., *S. flexneri*	Optical (colorimetric)	10^4^ CFU/ml	Mojarad and Gargaria, 2020
***Yersinia***							
N30yc5, N71yc2	Recombinant Yop51	**NR**	**NR**	**NR**	*NA*	*NA*	Bell et al., 1998
M1, M5, M7	Whole cell (*Yersinia entercolitica*)	Specific media (NaCl, beef extract, peptone, pH 7.2-7.4), 26°C	0.3 (L), 0.6 (E), 0.9 (PE)	*B. cereus, S. dysenteriae, L. monocytogenes, S. typhimurium, S. aureus, and E. coli*	*NA*	*NA*	Shoaib et al., 2020

NR, Not Reported, NA, Not applicable. *Extrapolated from culture conditions. ^*a*^Microbial culture conditions and growth conditions are listed for aptamer development only. The Microbial culture and growth conditions used for aptasensors development are listed in Supplementary Table S1. ^*b*^State: L, lag phase; E, exponential; PE, post-exponential. ^*c*^If number of strains used for counter selection is higher than to 5, they are listed in Supplementary Table S1.

## Aptamer Development

Aptamers are typically identified by SELEX (Systematic Evolution of Ligands through Exponential Enrichment). SELEX is an iterative process where repeated exposure of a target to a large pool of random oligonucleotides results in the gradual enrichment of specific sequences that bind with the highest affinity to the target. Since the technique’s inception in 1990, many variations of the original SELEX method have been published (Darmostuk et al., 2015). These experimental variations differ based on desired aptamer properties and details have been reviewed elsewhere (Wang et al., 2019). Of note, cell-SELEX can be used to select aptamers against whole cells in solution, to ensure cell surface target epitopes are in their native state (Kaur, 2018). This method is particularly useful for developing aptamers to detect water-borne pathogens. Cell-SELEX may include counter-selection steps to remove sequences binding to non-target microbes thus minimizing cross-reactivity and improving the specificity of the resulting aptamers (see Table 1 for examples).

**FIGURE 1:**
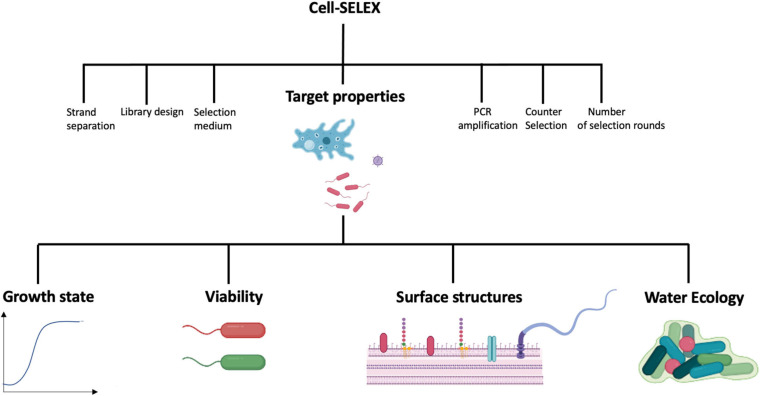
Factors affecting Cell-SELEX and thus the efficiency of aptamers targeting water-borne microbial pathogens. Most factors have been reviewed elsewhere, except the properties of the target, which are the topics of this review. Images were created in Biorender (https://biorender.com).

Several aptamer-coupled biosensing systems or aptasensors have been described for the detection of water-borne pathogens or toxins accumulating in water (Table 1 and Supplementary Table S1) with the majority targeting bacterial pathogens. Nevertheless, none have been officially adopted for routine detection of water-borne pathogens. The development of successful aptamer-coupled biosensors to detect water-borne pathogens requires a multi-pronged approach. Besides intricate knowledge of the sensing system, its transducer, the physico-chemical phenomenon that mediate signal responses, and a deep understanding of aptamer chemistries, careful consideration of the physiology and ecology of the target microorganism is required. This is because physio-ecological factors affect microbial morphologies and surface structures and thus the presence of aptamer targets (Figure 1). Although several works discuss transducing systems and aptamer design and chemistries in detail, relatively fewer studies consider the physio-ecological context of water-borne microbes for sensing platforms. Since most aptamers and aptasensing systems described in the literature detects water-borne bacterial pathogens, properties of bacteria are discussed in more detail to illustrate the importance of considering the target’s microbial characteristics for aptamer and aptasensor development.

## Aptamers Targeting Microbes in Specific States and Growth Conditions

Protozoan microbes have varying life cycles which can alternate between metabolically active feeding states, i.e., trophozoites, or inactive, dormant states such as oocysts or cysts (Aguilar-Díaz et al., 2011; Jain et al., 2019). Both oocysts and cysts are infectious forms that persist for long periods of time in environmental waters and resist a wide range of stressors (Omarova et al., 2018). The *Cryptosporidium parvum* oocyst-specific aptamer R4-6 was thus developed using cell-SELEX (Table 1; Iqbal et al., 2015). A counter selection step against *Giardia duodenalis*, another protozoan commonly found in water samples (Ongerth, 2013; WHO, 2017) was included to enhance aptamer specificity. This aptamer was first used in multiple assay formats on electrochemical biosensing platforms to detect oocysts of *C. parvum* down to 50 oocysts in river and lake water samples (Iqbal et al., 2015; Iqbal et al., 2019). Recently, a fluorescence plate assay coupled with magnetic beads labeled with a truncated version of the aptamer R4-6, named Min_Crypto2 achieved a detection limit of 5 oocysts (Hassan et al., 2021). The low LOD of this system is promising for oocyst detection in water given that the infectious dose of *C. parvum* is between 10 and 30 oocysts (Jain et al., 2019). Aptamer Min_Crypto2 was selective for *Cryptosporiudum* species, despite differences in size amongst species, but did not bind to *Giardia* oocysts. These features combined with its robust performance in water samples highlights its potential for oocyst detection in water.

Bacteria suspended in water are in a different metabolic state than bacteria growing in laboratory media. For example, *L. pneumophila* adopts a specific regulatory program when suspended in water due to starvation (Li et al., 2015). Consequently, the aptamers R10C5 and R10C1 were created by cell-SELEX using *L. pneumophila* suspended in water for 24 h, to allow the bacterium to adopt the associated metabolic state (Table 1). Counter selection was performed on two *Pseudomonas* spp. strains, prevalent in environmental waters (Paranjape et al., 2020). Both aptamers have excellent specificity for *L. pneumophila* (Saad et al., 2020).

Water borne bacteria can also be biofilm-associated. These bacteria can gain adaptive traits which make it harder to eliminate or disinfect them. To that end, biofilm-derived *Pseudomonas aeruginosa* cells were used to select aptamers through Cell-SELEX, without counter selection (Soundy and Day, 2017). The resulting aptamers were specific for 4 out of 5 clinical *Pseudomona*s *aeruginosa* isolates, minimally labeled non-*Pseudomonas* bacteria, and bound to both biofilm derived and planktonic *Pseudomonas* cells. The authors created chimeras and generated aptamers St17Lp21, St21Lp17. The chimeric aptamers showed improved binding and enhanced specificity for *Pseudomonas* bacteria as compared to the parent non-chimeric aptamers but were still unable to differentiate between biofilm and planktonic cells. This is not surprising since the biofilm-derived cells were washed and vortexed to release cells and remove alginate and exopolysaccharides. Mechanical stress induced by vortexing can destroy larger surface structures such as fimbriae and flagella. The lack of counter-selection coupled with the vigorous washing steps may have exposed cell surface structures not unique to the biofilm-derived *Pseudomonas*. Using counter selection could have eliminated sequences that bind to surface structures such as LPS or ubiquitous OMPS that are common in both planktonic and biofilm-derived *Pseudomonas*.

Aptamers against *Yersinia enterocolitica* were generated using Cell-SELEX with bacteria grown at 26°C (Shoaib et al., 2020). After counter selecting with several bacterial pathogens, the three aptamers M1, M5, and M7 were isolated (Table 1). *Y. enterocolitica* grown at 37°C showed reduced binding by the aptamers compared to bacteria grown at 25°C. Presumably this aptamer is specific for a cell surface component mostly expressed at low temperature. This study illustrates another characteristic of bacteria, which are temperature dependent surface structure and morphological changes. In the case of *Y. enterocolitica* specifically, the bacterium inhibits flagellum synthesis at 37°C (Kapatral et al., 1996). Components of the LPS are also temperature regulated (Białas et al., 2012).

## Aptamers Targeting Viable Cells

The ability to differentiate between dead and viable cells has important implications when assessing the risk or hazard of a microbe. For example, it would be costly and inefficient to administer shutdowns or disinfection protocols for the presence of dead pathogens in a system. The detection of viable cells is also important to determine the efficacy of water disinfection protocols. Some aptamers are able to differentiate between live and dead cells. Aptamer 33, specific for *Salmonella enterica* serovar Typhimurium, does not bind heat-killed cells (Table 1; Joshi et al., 2009). This aptamer might therefore be useful for monitoring the efficiency of heat-based disinfection. This aptamer is described in more detail below. Another example is aptamer ONS-23 created against whole cell *C. jejuni* (Table 1; Dwivedi et al., 2010). This aptamer was developed, using cell-SELEX, against a chicken isolate showing characteristic *C. jejuni* morphology (Thomas et al., 2002). Given that *C. jejuni* is found on raw poultry as well as in the gastrointestinal tract and feces of animals (Mughini-Gras et al., 2016), 20 bacterial species were used for counter selection, including food-borne pathogens, enteric bacteria, non-enteric bacteria and lactic acid bacteria. ONS-23 is therefore highly specific to *C. jejuni* strains showing minimal binding to non-*C. jejuni* strains (Dwivedi et al., 2010). Furthermore, ONS-23 does not bind non-viable *C. jejuni* (Kim Y. J. et al., 2018) indicating that it is specific for a surface structure only present on live *C. jejuni* cells (Kim Y. J. et al., 2018). Though this aptamer was not tested for water application, its selective properties for viable *C. jejuni* makes it promising for monitoring disinfection processes.

## Aptamers Targeting Source-Or Application-Specific Isolates

Isolates that are representative of the sample source of the downstream application should be used during aptamer development to ensure usefulness of the aptasensor. Aptamers E1, E2, and E10 were generated against a non-pathogenic *E. coli* strain of fecal origin (Crooks strain) using cell-SELEX (Table 1; Kim et al., 2013). For counter selection a combination of fecal coliform species and two Gram positives were used. The resulting aptamers were better at binding *E. coli* isolates of fecal origin than others and showed low binding to other species including laboratory strains of *E. coli* (Kim et al., 2013; Jin et al., 2017; Wu et al., 2017). A detection system using aptamer E2 was able to detect the Crooks strain in spiked tap water, pond water and milk, making it promising for *E. coli* detection in water (Jin et al., 2017).

## Aptamers Targeting Specific Surface Structures

Surface structures can be differentially expressed in response to growth states and environment (Justice et al., 2004; Van Der Woude and Bäumler, 2004; Liu et al., 2012; Fonseca and Swanson, 2014; Li et al., 2015). If the aptamer surface target is not differentially regulated then aptamers may bind cells in several conditions, including exponential and post-exponential phase. Examples of these are the ST2P aptamer against whole cell *S. enterica* Typhimurium (Duan et al., 2013b, 2014, 2016) and the *E. coli* E2 aptamer (Kim et al., 2013; Jin et al., 2017; Wu et al., 2017). Instead of whole cells, surface structures related to virulence can also be used as aptamer targets. The pathotype EHEC (*E. coli* enterohemorrhagic) contains the infamous O157:H7 serotype which is strongly linked to deadly outbreaks from contaminated drinking water (Solomon et al., 2002; Ali, 2004; Saxena et al., 2015). For detecting this serotype, the specific variant of LPS can be exploited. *E. coli* aptamers a-aptamer and c-aptamer were created against LPS of *E. coli* O157:H7 (Table 1; Bruno et al., 2009). These aptamers were used in several aptasensing platforms to detect whole *E. coli* O157:H7 cells with great specificity, showing minimal signals with other serotypes (Wu et al., 2015; Díaz-Amaya et al., 2019a,b; Hao et al., 2019; Jiang et al., 2020). The aptamers could bind to heat-killed and formalin killed *E. coli* (Hao et al., 2019; Jiang et al., 2020). This is likely due to the fact that these treatments do not negatively affect the LPS (Gao et al., 2006; Chafin et al., 2013). This approach allowed for very specific aptamers to be developed; however, since the target persists after killing of cells, the aptamers are of limited use for monitoring the efficacy of disinfection programs in water. This illustrates the need for designing aptamers relevant to the downstream application.

Outer membrane proteins (OMP) of Typhimurium were used to create Aptamer 33. Counter selection was done with purified LPS of the *Salmonella* isolate as well as OMPs and LPS from *E. coli*. Aptamer 33 showed pan-serovar specificity, binding to seven different serovars of *S. enterica* in one study and four different *S. enterica* serovars in another study (Joshi et al., 2009; Hasan et al., 2018). The aptamer was used in a fluorescence aptasensor to detect whole Typhimurium in water samples from different sources highlighting its potential for detection in water (Duan et al., 2012). The aptamer does not bind to heat-killed Typhimurium which is to be expected as most OMPs are heat labile (Oh et al., 2017). The authors also observed that the aptamers could not bind *S.* enterica serovars Tennessee and Muenchen. This suggests that the aptamer may not have broad serovar specificity.

## Discussion

Aptamer-coupled biosensors are promising systems for the detection of pathogens in water samples but are limited in real-world applications. There are a few things to consider to improve aptamers practicality in aptasensing technology (Figure 1). Many studies do not explicitly report the growth states and conditions used during cell-SELEX or during subsequent testing of the aptamers (Table 1 and Supplementary Table 1). For example, OD_600_ values are meaningless without details about the growth conditions, including medium, temperature and aeration. We suggest that instead of reporting OD_600_, the growth phase should be determined and reported, as done by Zou et al. (2018), as this would offer insight into an aptamer’s potential for specific applications. Regardless, it is important to keep the end goal in mind while developing aptamers. For example, monitoring efficiency of disinfection program will require discerning viable cells from dead cells. Aptamer ONS23 and Aptamer 33 are able to distinguish between live and dead cells (Joshi et al., 2009; Dwivedi et al., 2010; Oh et al., 2017; Kim Y. J. et al., 2018). A cell-SELEX strategy for such an application could use dead cells for counter selection. Another factor to consider is the physio-morphological state of microbes. This ensures that the microbial target possesses traits and characteristics that are representative of what’s typically found in the environment that will be sampled. For example, biofilm-derived cells might be used (Soundy and Day, 2017), but care must be taken not to remove the biofilm-specific target when preparing the target for cell-SELEX. Alternatively, if the end goal is to detect pathogens in water, then bacteria suspended in water may be used as the target (Saad et al., 2020). Lastly, it is not trivial to select appropriate strains for counter selection. This will impact aptamer affinities for targets in source environments. A possible approach is to use a cocktail of strains for the target species and a cocktail of species typically found in the same environment for counter-selection (Dwivedi et al., 2010; Kim et al., 2013). In conclusion, it is necessary to better elucidate the microbial target and the limitation of its cognate aptamer to help push microbial aptasensing platforms to market. As such a collaborative effort is needed between academics and stakeholders (governments, industry, engineers) to develop both transducer and aptamer technologies for specific microbial contaminants in the context of source water, taking into account the particularities of the microbe and its physiological state.

## Author Contributions

MS reviewed the literature and compiled the information reported here, and wrote the first draft of the manuscript. MS and SPF edited the manuscript. Both authors approved submission of the manuscript.

## Conflict of Interest

The authors, together with Maryam Tabrizian (McGill University, Department of Biomedical Engineering), are the inventors of aptamers R10C1 and R10C5, subject of patent applications filed in United States, patent application number US 16/850,355; and in Canada – patent application number pending at the time of revised manuscript submission. At the time of submission of the manuscript, the applications were under review. The authors declare that the research was conducted in the absence of any commercial or financial relationships that could be construed as a potential conflict of interest.
